# Too Many Free Samples

**Published:** 1884-01-20

**Authors:** 


### *TOO MANY FREE SAMPLES.*

The free distribution of medical journals as samples is seriously affecting the business of journalism in the United States. A frequent reply to agents soliciting subscribers is that recently made to an agent of this Journal—"No, sir, I have no need to subscribe for any medical publication, as I am in almost daily receipt of sample journals from every section of the Union, which furnishes me with all the reading I want." Here is food for reflection. We opine that this subject would be one worthy of consideration at the next meeting of the Association of American Medical Editors.

